# ROS and Antioxidants: Prospective Therapeutic Options in Cancer

**DOI:** 10.1111/jcmm.71288

**Published:** 2026-07-14

**Authors:** Kostas A. Papavassiliou, Amalia A. Sofianidi, Angeliki Margoni, Athanasios G. Papavassiliou

**Affiliations:** ^1^ First University Department of Respiratory Medicine, ‘Sotiria’ Chest Hospital, Medical School National and Kapodistrian University of Athens Athens Greece; ^2^ Department of Biological Chemistry, Medical School National and Kapodistrian University of Athens Athens Greece

**Keywords:** antioxidants, biomarkers, cancer therapy, personalized medicine, reactive oxygen species (ROS)

## 1 Introduction

Reactive oxygen species (ROS) have been recognized as key regulators of cancer development and therapeutic response [1]. These molecules arise as metabolic byproducts during cellular processes, mainly within mitochondria through oxidative phosphorylation. Notably, in this regard, NADPH oxidases (NOXs) are also sources of ROS in tumour cells [2, 3].

In healthy cells, ROS act as crucial signalling mediators that govern cell proliferation, differentiation and metabolism, maintaining a balanced state known as oxidative eustress [4]. In contrast, cancer cells exhibit a distinct redox balance characterized by elevated ROS levels relative to normal cells, facilitating metabolic reprogramming and supporting hallmark features of malignancy [1]. Although this equilibrium is highly fragile—where moderate ROS levels promote tumour growth and dissemination, but excessive accumulation induces cell death—cancer cells adapt mechanisms that allow their survival under such conditions [1].

ROS contribute to tumour cell proliferation, angiogenesis, metastasis and genomic instability. To counteract oxidative stress, tumour cells often enhance their internal antioxidant systems, as excessive ROS can trigger apoptosis [5]. This duality creates a therapeutic dilemma: while antioxidants may theoretically inhibit tumour progression by limiting oxidative DNA damage, they might simultaneously shield cancer cells from ROS‐induced cytotoxicity. A major concern is that antioxidant supplementation could reduce the effectiveness of anticancer therapies that depend on oxidative damage for their action [6]. Accordingly, the National Comprehensive Cancer Network (NCCN) advises against antioxidant use during chemotherapy, radiotherapy, or photodynamic therapy [7]. Clinical evidence supports this caution. For instance, the SWOG S0221 trial reported a higher risk of recurrence and mortality among patients taking antioxidant supplements during chemotherapy [8]. Likewise, findings from the MARIE study associated concurrent antioxidant use with increased mortality and poorer recurrence‐free survival [9]. Despite promising preclinical data supporting antioxidant‐based strategies and combination therapies [10], successful clinical application remains limited. There is a pressing need for more effective approaches to optimize antioxidant use in oncology and improve patient outcomes.

## 2 Toward Precision Antioxidant Therapy: Biomarkers and Personalization

Personalized medicine represents a promising strategy for integrating antioxidants into cancer treatment. Tumours display significant heterogeneity both within and between individuals, posing a major challenge in oncology [11]. This variability is influenced by components of the tumour microenvironment (TME), including cancer‐associated fibroblasts (CAFs) and immune cells [11]. Moving beyond uniform supplementation strategies toward individualized approaches based on genetic, metabolic and redox characteristics is increasingly attractive [12]. Oxidative stress levels differ substantially across tumour types. For example, melanoma is a well‐established ROS‐driven malignancy, where ultraviolet radiation induces oxidative stress in melanocytes [13]. Lung cancers, including squamous cell carcinoma and adenocarcinoma, also exhibit high oxidative burden, often associated with mutations in the Kelch‐like ECH‐associated protein 1 (KEAP1)–nuclear factor erythroid 2‐related factor 2 (NRF2) pathway, a key transcriptional regulator of cellular antioxidant defences [14]. These mutations suggest adaptation to oxidative environments by altering antioxidant capacity. Future strategies may focus on tumours with elevated ROS levels, aiming to modulate the TME. Additionally, patients with low baseline antioxidant status may benefit from supplementation and measuring circulating antioxidant levels could help identify suitable candidates [15].

Identifying oxidative stress‐related biomarkers offers another avenue for personalized treatment. Genetic variability influences therapeutic responses, and recent studies have shown that polymorphisms in oxidative stress‐related genes contribute to treatment resistance, particularly in hematologic malignancies [16]. Such genetic markers could help predict which patients may benefit from antioxidant therapy. For example, a prognostic antioxidant gene signature has been identified in kidney renal clear cell carcinoma, where high‐risk patients exhibit increased immune activity within the TME. In such cases, combining antioxidants with immunotherapy may be advantageous [17]. Moreover, monitoring oxidative stress biomarkers can help assess treatment response [18]. Artificial intelligence (AI) is increasingly important in identifying such biomarkers [19]. Supporting this approach, studies have demonstrated that *N*‐acetylcysteine (NAC) and vitamin C effectively reduce tumour growth in B‐cell lymphomas with high MYC expression, but not in tumours with low MYC levels. This highlights MYC as a potential biomarker for targeted antioxidant therapy in refractory/relapsed B‐cell lymphomas [20].

## 3 Therapeutic Strategies to Optimize Antioxidant Use in Cancer

While these concepts provide valuable theoretical insights, practical implementation remains challenging. Beyond personalization, improving antioxidant formulations is essential. Conventional antioxidants often suffer from poor bioavailability and instability. Nanotechnology offers promising solutions, enabling targeted delivery, controlled release and protection from degradation [21]. For example, nanoparticle‐based delivery of silymarin has shown enhanced anticancer effects in colorectal cancer models [22]. Similarly, nanoparticle formulations combining curcumin with chemotherapeutic agents have demonstrated efficacy in pancreatic cancer cells [23]. However, translating these innovations into clinical practice remains difficult due to manufacturing complexity, unpredictable biological responses and cost barriers. Integration of AI and machine learning in nanoparticle design may accelerate optimization, improve safety profiles and reduce development costs [24].

Optimizing the timing and dosage of antioxidant administration is equally critical. Emerging evidence suggests that high‐dose intravenous vitamin C may exert anticancer effects by acting as a pro‐oxidant, enhancing chemotherapy efficacy while reducing toxicity [25]. However, such findings are largely confined to preclinical studies. Advances in pharmacokinetics and pharmacodynamics could help refine dosing strategies. AI has the potential to revolutionize drug development by integrating diverse datasets from chemical, biological and clinical sources [26].

Antioxidants may also enhance the efficacy of existing anticancer therapies. For instance, antibody‐drug conjugates (ADCs) are susceptible to oxidative degradation, and antioxidant excipients such as sucrose, methionine and ascorbic acid have been used to improve their stability [27]. Beyond formulation, antioxidants can be co‐administered therapeutically. Vitamin C, when delivered via nanoparticles, can induce oxidative damage and promote macrophage polarization, enhancing immune activation within the TME [28]. Similarly, vitamin E has been shown to improve outcomes when combined with immune checkpoint inhibitors (ICIs) by restoring dendritic cell function via targeting Src homology region 2 domain‐containing phosphatase 1 (SHP1) protein [29]. In targeted therapies, antioxidants may help overcome resistance. Curcumin, for example, has been shown to increase the sensitivity of non‐small cell lung cancer (NSCLC) to the tyrosine kinase inhibitor (TKI) crizotinib [30, 31]. Since curcumin has antioxidant and pro‐oxidant activities [32, 33, 34], the effects on NSCLC cells may arise from its pro‐oxidant potential. Likewise, the use of certain flavonoids in cancer therapy may be based on their pro‐oxidant activities.

The phosphoinositide‐3‐kinase (PI3K)/AKT (PKB) serine–threonine kinase/mechanistic target of rapamycin kinase (mTOR) signalling pathway is closely linked to oxidative stress, with bidirectional interactions. Human epidermal growth factor receptor 3 (HER‐3) signalling, a key oncogenic pathway, is not effectively inhibited by current TKIs [35]. TKI treatment increases ROS levels, which can inhibit protein tyrosine phosphatases (PTPs) and alter signalling dynamics. Interestingly, combining TKIs with antioxidants may suppress resistant HER‐3 signalling [36]. Novel HER‐3‐targeting ADCs currently under clinical investigation could potentially be combined with antioxidant strategies [37].

A significant challenge in cancer therapy is ROS‐mediated chemoresistance. Resistant cancer cells often exhibit both elevated ROS levels and enhanced antioxidant defences, particularly via the KEAP1–NRF2 pathway, glutathione and thioredoxin systems. This has been observed across multiple chemotherapeutic agents. Targeting these antioxidant systems, through glutathione depletion, NRF2 inhibition, NOX inhibition, or modulation of mitochondrial and endoplasmic reticulum (ER) stress, may improve treatment outcomes [38]. Enzymatic antioxidants combined with chemotherapy are currently being evaluated in clinical trials. In addition to the KEAP1–NRF2 signalling pathway, other transcription factors, including nuclear factor kappa‐light‐chain‐enhancer of activated B cells (NF‐κB), peroxisome proliferator‐activated receptor gamma coactivator 1 alpha (PGC‐1α), forkhead box O (FOXO) transcription factors and hypoxia‐inducible factor‐1 alpha (HIF‐1α), also contribute to ROS‐related resistance [38].

At the molecular level, transcription factors are central regulators of cancer progression and represent promising therapeutic targets [39]. Because they integrate oncogenic signalling and control gene expression, their inhibition may effectively suppress tumour growth while limiting resistance mechanisms. Combining transcription factor‐targeting therapies with antioxidants may enhance clinical efficacy [39]. Among these, NRF2 and BTB domain and CNC homology 1 (BACH1) transcription factors are key regulators of antioxidant responses [40]. NRF2 expression may serve as a biomarker for antioxidant therapy, indicating tumours with high antioxidant capacity [41]. Targeting NRF2, especially alongside pro‐oxidant antioxidant strategies, may yield significant therapeutic benefits. Table 1 presents a summary of the proposed therapeutic strategies to improve antioxidant supplementation in patients with cancer.

**TABLE 1:** Context‐dependent strategies for the rational use of antioxidants in cancer.

Strategy	Core concept	Clinical relevance	Key limitation
Personalized, biomarker‐guided use	Stratify patients by redox status, genetic signatures (e.g., MYC, KEAP1/NRF2) and antioxidant levels	Enables selective benefit in defined subgroups; avoids indiscriminate supplementation	Lack of validated biomarkers; tumour heterogeneity
Redox modulation (dose/timing optimization)	Exploit context‐dependent antioxidant vs. pro‐oxidant effects (e.g., high‐dose vitamin C)	May enhance therapeutic efficacy and reduce toxicity	Mostly preclinical; unclear dosing and scheduling
Combination with anticancer therapies	Integrate with chemotherapy, immunotherapy, targeted agents and ADCs	Potential to improve efficacy, stability and immune response	Risk of protecting tumour cells; inconsistent clinical evidence
Targeting redox‐adaptive resistance	Inhibit NRF2, glutathione, thioredoxin systems; disrupt ROS homeostasis	May restore chemosensitivity in resistant tumours	Toxicity risk; complex redox network; limited clinical validation
Transcription factor targeting	Modulate NF‐κB, PGC‐1α, FOXO, HIF‐1α, NRF2, BACH1 pathways	Upstream control of tumour survival and oxidative stress responses	Difficult drug targeting; off‐target effects
Advanced delivery systems	Nanoparticle‐based targeting and controlled release	Improves bioavailability and tumour specificity	Manufacturing complexity; translational barriers
AI‐assisted optimization	Use machine learning for biomarker discovery and treatment design	Supports precision oncology and accelerates development	Data quality, validation and implementation challenges

Abbreviations: ADCs, antibody‐drug conjugates; AI, artificial intelligence; BACH1, BTB domain and CNC homology 1; FOXO, forkhead box O; HIF‐1α, hypoxia‐inducible factor‐1 alpha; KEAP1, Kelch‐like ECH‐associated protein 1; MYC, MYC proto‐oncogene; NF‐κB, nuclear factor kappa‐light‐chain‐enhancer of activated B cells; NRF2, nuclear factor erythroid 2‐related factor 2; PGC‐1α, peroxisome proliferator‐activated receptor gamma coactivator 1 alpha; ROS, reactive oxygen species.

## 4 Conclusions—Future Perspectives

In summary, the role of antioxidants in cancer therapy is highly context‐dependent. Although they may contribute to tumour progression in certain settings, they can be beneficial in specific cancers such as MYC‐driven lymphomas and immunotherapy‐responsive tumours. Their effective use depends on personalized approaches considering tumour type, stage, genetic background and microenvironment. Combining antioxidants with existing therapies may help overcome resistance and improve outcomes. However, precise targeting is essential to maintain redox balance without harming normal cells. Advances in nanotechnology and AI‐driven biomarker identification may further refine antioxidant strategies. Until more definitive clinical evidence becomes available, caution is warranted, and antioxidant supplementation during cancer treatment should generally be avoided.

## Author Contributions

**Kostas A. Papavassiliou:** conceptualization, data curation, writing – original draft. **Amalia A. Sofianidi:** data curation, writing – original draft. **Angeliki Margoni:** writing – original draft. **Athanasios G. Papavassiliou:** conceptualization, data curation, supervision, writing – review and editing.

## Funding

The authors have nothing to report.

## Conflicts of Interest

The authors declare no conflicts of interest.

## Data Availability

Data sharing is not applicable to this article as no datasets were generated or analysed during the current study.
